# Emotional-psychological concerns of Turner’s patients regarding social discrimination

**DOI:** 10.1192/j.eurpsy.2023.1882

**Published:** 2023-07-19

**Authors:** N. Bouayed Abdelmoula, B. Abdelmoula, S. Sellami

**Affiliations:** Genomics of Signalopathies at the service of Medicine, Medical University of Sfax, Sfax, Tunisia

## Abstract

**Introduction:**

Turner syndrome characterized by total/partial and/or homogeneous/mosaic X chromosome monosomy is associated with various physical health concerns, including facial dysmorphism, short stature, infertility, and other organ defects such as heart, kidney, bone, skin, … as well as variable degrees of cognitive impairments. Besides, social skills, communication and relationships are usually disordered.

**Objectives:**

The aim of this study was to report social challenges of Turner syndrome particularly related to discrimination regarding physical and cognitive impairments.

**Methods:**

A retrospective analysis of clinic data and karyotypes were carried out for the patients diagnosed with Turner syndrome among patients who consulted at our genetic counselling at the medical University of Sfax, during the last two decades.

Cytogenetic analysis were carried out using conventional methods and RHG banding with analysis of at least 20 metaphases and 3 karyotypes for each patient. Social challenges were recorded for each patient during pre-cytogenetic consultation and oriented questioning.

**Results:**

We identified 23 cases referred with a cytogenetic diagnosis of monsomy X. The karyotyping was indicated for dysmorphism, primary or secondary amenorrhea, female infertility and recurrent pregnancy losses. Homogeneous X chromosome monosomy was recorded in 13% of cases, whereas mosaic forms with and without structural X/Y abnormalities were more frequent (82%). The mean age of patients in the study was twenty years. When the 45,X population was the predominant one (56,5%), dysmorphism and primary amenorrhea were constant. In the mosaic forms, clinical traits of Turner syndrome were insignificant. Discrimination based on physical appearance, intellectual disability, and failure to conceive were the three types of social challenges revealed by patients of our study. Parents of Turner patients were also concerned at the psychological level. They in fact revealed their emotional distress face to stressful experiences of their children with Turner syndrome regarding the social discrimination they encountered particularly in schools.

**Conclusions:**

Social challenges related to discrimination based on physical appearance, intellectual disability, and failure to conceive in Turner syndrome lead to depression, low self-esteem and anxiety.

**Disclosure of Interest:**

None Declared

